# Recent progress in pig-to-human kidney xenotransplantation

**DOI:** 10.3389/fimmu.2025.1735113

**Published:** 2025-12-10

**Authors:** Pei Tao, Kaiyu Zhou

**Affiliations:** 1 Department of Pediatric Pulmonology and Immunology, West China Second University Hospital, Sichuan University, Chengdu, China; 2 Chengdu Women and Children’s Central Hospital, School of Medicine, University of Electronic Science and Technology of China, Chengdu, Sichuan, China

**Keywords:** xenotransplantation, porcine kidney, graft survival, genetic engineering, organ shortage, transplantation immunology

## Abstract

Even though the patient kept up his dialysis regimen as he had before xenotransplantation, this is a significant development for xenotransplantation. This benchmark for xenotransplantation has the following significant findings. Firstly, from the patient’s perspective, the patient was able to regain energy and quality of life and experience normal metabolic filtration (creatinine clearance) during the functional period, thanks to the transplant, which significantly reduced his need for dialysis. Secondly, this is a historic accomplishment for xenotransplantation because it set up a new global record for xenotransplantation survival that exceeds all prior attempts. Thirdly, it brings hope for solving the problem of worldwide organ shortage. Lastly, the FDA has authorized more extensive clinical trials that may include more than thirty patients from several transplant facilities.

## Introduction

A significant advancement in xenotransplantation has been demonstrated by the recent case of a porcine kidney that functioned in a human for a record-breaking 271 days before being removed due to continuous proteinuria (1). The donor is an eGenesis 69 gene-edited pig. Before this, eGenesis Inc. produced porcine endogenous retroviruses (PERV)-free swine (2, 3). After that, they proceeded with more gene editing, which involved deleting three glycan antigens, inserting seven human transgenes, and inactivating PERV (A, B, and C) (4). Ten of the 69 genes are edited to inhibit blood clotting and immunological rejection. These ten gene modifications include: knocking out three xeno-antigens (GTKO/β4GalNT2/CMAH), and inserting seven human transgenes for immune regulation (CD46, CD55, EPCR, TBM, CD47, HO1, A20). Based on previous publication, in order to induce immunosuppression, this patient received anti-thymocyte globulin (ATG), anti-CD20 antibody, and C3 inhibitor for immunosuppressant induction, and an Fc silent anti-CD154 agent, tacrolimus, mycophenolate mofetil, and steroids for maintenance (5).

## Mechanisms of graft survival and rejection

For the short-term graft survival and function, it is related to the elimination of hyperacute rejection (HAR). These were the efforts of “three Davids”, who are David JG White (6–10), David H Sachs (11–13), and David KC Cooper (10, 14), with their contribution of xenografts of kidney, heart, and islet to nonhuman primates. They found that the α-Gal antigen contributes to HAR. Therefore, multi-gene editing effectively neutralized the three principal xenoantigens (GTKO/β4GalNT2/CMAH), and transgenic expression of human complement-regulating proteins (CD46, CD55) inhibited complement-mediated hyperacute rejection, as seen by the uneventful immediate post-operative period. This eliminates the main obstacle to clinical xenotransplantation that had existed for many years. Moreover, in earlier attempts to alleviate molecular incompatibilities in the coagulation cascade, transgenic expression of thrombomodulin (TBM), endothelial protein C receptor (EPCR), and CD47 minimized the thrombotic microangiopathy (TMA) that led to early graft failure. Further, in contrast to traditional CNI-based regimens, the induction regimen (anti-thymocyte globulin, anti-CD20 antibody) in conjunction with maintenance by an Fc silent anti-CD154 agent costimulatory blocking therapy effectively circumvented acute cellular rejection during the crucial first month. Therefore, the patient experienced immediate graft function without requiring extended dialysis.

## Clinical outcomes and field overview

The 271-day timetable is approximately in accordance with NHP results, which demonstrate that chronic antibody-mediated rejection (AMR) (15) and TMA eventually become the predominant failure mechanisms even with ideal genetic modification (16, 17). Research utilizing comparable 3KO/7-transgene kidneys in macaques revealed that 176 days is the median survival, with a maximum of more than two years (16, 17). Further, interstitial fibrosis and progressive AMR, TMA were common in long-term survivors of grafts that lasted more than six months (16, 17). Alloantibody production progressively overcame complement regulation in the human example, which most likely followed this course. This study has significant clinical translation promise. The patient was able to engage in regular activities during the approximately nine months of graft function, which resulted in meaningful dialysis-free survival. Even though long-term function is still problematic, this proves that xenotransplantation is a feasible bridging therapy. The functional length is getting close to the 12-week maximum specified for delayed recovery of graft function in allotransplantation.

Transplanting organs from one species to another, called xenotransplantation, has long been seen as a viable remedy for the organ scarcity issue (18). Human organ shortages are still a major problem, with thousands of people waiting for life-saving kidney transplants globally. We are now closer to an effective replacement thanks to the recent success of kidney transplantation from pigs to humans (19, 20). Before this progress, there were several clinical cases of porcine kidney xenotransplantation from the Robert A Montgomery group, New York University Langone Health (21, 22), and the Jayme E Locke group, University of Alabama at Birmingham (23). All of these studies using 10 gene-edited porcine from the United Therapeutics, and these groups perform delicate studies covering immune responses (24, 25), cellular dynamics (26), and multiomics (27, 28). Therefore, the United Therapeutics and eGenesis-led clinical studies for patients with end-stage kidney disease were approved by the U.S. Food and Drug Administration in 2025. It is also the efforts of the International Xenotransplantation Association (IXA), including the past, present, and in-elect president, councilors (29–31), and members, especially Leo Buhler (32–34), Wayne J Hawthorne (33, 35), Muhammad Mohiuddin (the first porcine heart transplantation to human patients) (18, 36–38), Burcin Ekser (39), Joe Tector (40–42), Jay Fisherman (34), and so on (18, 43, 44).

Recent developments in immunosuppressive treatments and genetic engineering have played a major role in the success of xenotransplantation. To lower the possibility of immunological rejection, scientists have created genetically modified swine with several genetic changes. For example, the pig used in the recent transplant underwent ten genetic changes, including the insertion of human genes to protect the transplanted organ and the silencing of genes that generate antigens that cause human immune reactions. Knock-out and knock-in gene editing are both included in the 10 gene editing. GGTA1, β4GalNT2, and CMAH are all knocked out to stop the kidney from growing excessively (45). Complement inhibitor genes include human CD46 and human DAF (also called CD55) (6, 46). It may be possible to prevent complement activation by knocking in these two genes. Human endothelial C receptor (EPCR)and human thrombomodulin (TBM) are two additional genes that can be knocked in to stop microscopic blood clots (thrombotic microangiopathy) (47). To mitigate inflammation in the xenograft, human hemeoxygenase-1 (HO1) and CD47 are knocked in. HO1 possesses potent anti-inflammatory, anti-oxidative, and anti-apoptotic properties. When a cell is injured or infected, it undergoes apoptosis, which is programmed cell death. The activation of phagocytic macrophages and T cell infiltration is inhibited by CD47 (48). Procines from both eGenesis and Revivicor had these 10 gene modifications.

Apart from these reported cases, another ongoing xeno-kidney clinical trial is being performed in China. In this study, a Chinese female patient aged 69 received the xeno-kidney transplantation at Xi’jing Hospital, led by the group of Dr. Kefeng Dou. The gene-edited pig kidney is from ClonOrgan, and to date, the porcine kidney has still been functioning well for almost eight months. In this case, the porcine is 6-gene edited. In Bama miniature pigs, a 6-gene-edited porcine model was generated by concurrently knocking out three key xenoantigens (GGTA1, CMAH, and β4GalNT2), inserting the anti-coagulation factor THBD, and adding two complement regulatory proteins (hCD55 and hCD46) (49). Previously, Kefeng Dou’s group utilized this 6-gene-edited porcine liver to perform the first case of xeno-liver transplantation (50–52). This case of 6-gene-edited miniature porcine kidney xenotransplantation to human patients brought us hope that longer survival of xeno-kidney could be achieved in the near future.

## Current challenges and future directions

However, there still exist multiple challenges for xenotransplantation. First of all, there is a therapeutic paradox. Intensive immunosuppression, which is necessary for long-term survival, would increase the risk of infection, while reduced immunosuppression brought on by infections would lead to rejection. Secondly, even though the initial creatinine clearance was strong, the ultimate deterioration indicates a pathophysiology similar to progressive chronic allograft nephropathy (CAN). This is similar to the long-term results of allotransplants, when years of inevitable function loss are caused by multifactorial scarring processes. But xenografts seem to shorten this time frame, as fibrotic alterations can be seen histologically in months as opposed to years (53). Thirdly, can AMR be prevented beyond 1 year? There are several trials on thymus-kidney transplantation at CCTI of Columbia University to induce tolerance (54). Further, to date, the patient who received a 6 gene-modified porcine kidney has achieved survival of more than 8 months. Hopefully, the transplanted kidney could have a prolonged function than previous studies. Fourthly, could less genetic modification be the potential solution for longer survival? There might be some compensation mechanism with this genetic modification. Otherwise, the grafts would not be a loss of function for longer than 9 months. For example, the patient from Xi’jing Hospital, China, has survived for more than 8 months, with less genetic modification (a 6 gene-edited porcine kidney). Lastly, the incorporation of multi-omics of the 271 surviving porcine kidney needs to be performed to unravel the mechanisms that lead to chronic AMR.

This successful case has broader clinical translation. It provides a critical proof-of-concept that multi-gene editing (69 modifications) can achieve extended survival. Furthermore, with delicate immunosuppression management, hyperacute rejection could be prevented. Moreover, the quality of the patient’s life can be meaningfully improved even with temporary graft function. Lastly, the 9-month window offers valuable data on long-term immune responses and graft adaptation.

## Conclusion

Overall, despite these opening questions, an important breakthrough in the area of xenotransplantation has been made with the recent success of a kidney transplant from a 69 gene-edited porcine that survived for 271 days in a human recipient. It draws attention to the possibility of using genetically modified porcine as a source of organs for transplantation into humans. Moreover, the approximately 8 months of survival of xeno-kidney transplantation in China brings us hope for the future of organ availability because of the progress that has been made thus far. To guarantee that xenotransplantation can safely and successfully satisfy the demands of patients waiting for life-saving organ transplants, additional investigation is still needed.

## Data Availability

The original contributions presented in the study are included in the article/supplementary material. Further inquiries can be directed to the corresponding author.
